# Ramadan and diabetes - knowledge, attitude and practices of general practitioners; a cross-sectional study

**DOI:** 10.12669/pjms.324.9904

**Published:** 2016

**Authors:** Muhammad Yakoob Ahmedani, Bella Z. Hashmi, Muhammad Saif Ulhaque

**Affiliations:** 1 Muhammad Yakoob Ahmedani, FCPS. Professor of Medicine, Department of Medicine, Baqai Institute of Diabetology and Endocrinology Baqai Medical University, Karachi, Pakistan; 2 Bella Z. Hashmi, MBBS. Trainee Postgraduate (FCPS-II), Department of Medicine, Baqai Institute of Diabetology and Endocrinology Baqai Medical University, Karachi, Pakistan; 3 Muhammad Saif Ulhaque, MS (Diab & Endo). Registrar, Department of Medicine, Baqai Institute of Diabetology and Endocrinology Baqai Medical University, Karachi, Pakistan

**Keywords:** Diabetes, Ramadan, Fasting, General practitioners

## Abstract

**Background and aims:**

Fasting during Ramadan is obligatory for all Muslims across the world. Through literature review, it has been found out that there are various articles published for the awareness of patients and general population regarding safe fasting during Ramadan. But very few studies highlight the Ramadan specific knowledge of general practitioners engaged in providing care to people with diabetes. This study aims to describe the practice, knowledge and attitude of general practitioners regarding treatment and dietary modifications for people with diabetes during Ramadan across Pakistan.

**Methods:**

A cross-sectional descriptive study was undertaken among a sample of 274 general practitioners. Data was collected by means of a questionnaire that consisted of 25 questions that were structured according to three categories i-e. Ramadan specific knowledge, diet and physical activity and treatment modification related knowledge and practices of GPs.

**Results:**

Out of the total population of GPs surveyed, 70% responded correctly to the questions while 30% responded incorrectly. 1/4^th^ of GPs incorrectly responded to questions regarding basic concepts of diabetes and Ramadan. 1/3^rd^ of GPs responded incorrectly regarding questions on diet.

Almost 40% of the GPs responded incorrectly to the questions regarding drug dosage adjustment in people with diabetes during Ramadan. However, more than 80% responded in agreement regarding alteration in medication timings.

**Conclusion:**

Almost one third of the studied populations of general practitioners across Pakistan lack the knowledge of basic principles that are important to be employed in the management of diabetes during Ramadan. Hence there is need to promote educational programmes and CMEs to improve the knowledge of our GPs that should be reflected by their sound clinical practices in the field of diabetes.

## INTRODUCTION

Fasting during the month of Ramadan is one of the five pillars of Islam. It is obligatory for all healthy adult Muslims to abstain from eating and drinking from pre-dawn to dusk during the whole month of Ramadan.^1^ However, there is no bar on food, drugs or fluid intake after sunset. During this entire month all the Muslims of the world are obligated to follow the recommended practices specified for this holy month. Around 50 million people with diabetes fast annually during the month of Ramadan if their diabetes is uncomplicated.^2^ Among people with diabetes, there are some at higher risk of complications during fasting while others can safely fast without major complications.^3^ Doctors trained in Ramadan specific diabetes education can identify people who are at higher risk of complications during fasting. They can also help them and guide in making decision to fast or not to fast and how to fast safely during Ramadan. Training of doctors is even more important in those countries where non-Muslim doctors have Muslim patients with diabetes who want to fast. It is imperative for health care providers to be aware of health and cultural (religious) practices of their patients and to educate them regarding these issues.

There are studies regarding assessment of Ramadan specific diabetes education level of patients^4^,^5^ but none of the studies have assessed education level of general practitioners in this regard. People with diabetes who want to fast may have poor knowledge, misconceptions, attitudes and improper practices followed during Ramadan. These people may not visit their doctors before Ramadan. On the other hand, doctors and other health care providers may not be geared up to guide their patients accordingly who wish to fast.

This study was undertaken to assess Ramadan specific education level of general practitioners managing people with diabetes across Pakistan. This study was a part of Ramadan specific education programme that was designed for general practitioners to educate them about Ramadan specific recommendations for people with diabetes.

## METHODS

This study was conducted by Ramadan study group of Baqai Institute of Diabetology & Endocrinology (BIDE) in the year 2013. Questionnaire based data was collected from general practitioners across 12 main cities of the country including Karachi, Hyderabad, Lahore, Multan, Faisalabad. Series of lectures as part of pre-Ramadan training workshops were organized to educate general practitioners regarding Ramadan and diabetes. Questionnaire included 25 questions to assess knowledge, attitude and practices of GPs regarding Ramadan and diabetes.

### Data Analysis

Data was entered on MS Excel and then transferred to SPSS for analysis. Percentages are calculated for every correct and incorrect answer of each question and are shown in the frequency table and graphs in the section of results.

## RESULTS

Study shows that 16.94% of the GPs responded that people with diabetes should never fast while 25.72% of the GPs said that taking insulin injection during fasting breaks the fast.6.4% of the GPs were of the view that Sehri can be skipped during Ramadan.19.02% of the GPs said that checking blood sugar levels during fasting is prohibited while 32.08% of GPs did not know about the proper action that should be advised to the patients if blood sugars level is 70mg/dl during the early hours of fasting. Almost 13.88% of the GPs said that strenuous exercise and physical activity is not hazardous during fasting in Ramadan.

Total numbers of 250+ GPs across the country were surveyed. Overall 70% responded correctly while 30% gave incorrect answers (Fig-I) 1/4^th^ of GPs responded incorrectly regarding basic concepts about diabetes and Ramadan (Fig.II). Around 1/4^th^ of the GPs answered incorrectly regarding the questions on physical activity and diet during Ramadan (Fig-III).

**Fig-1:**
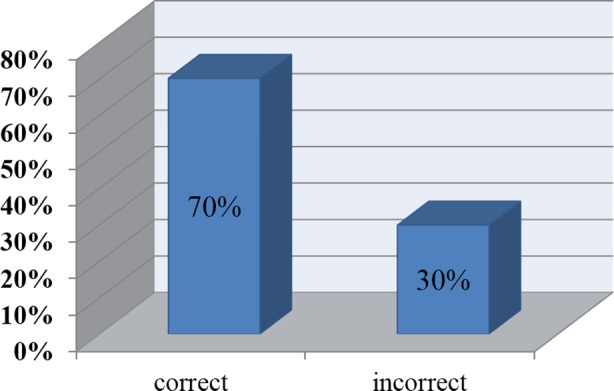
Graphical representation of overall result of Ramadan specific knowledge of general practitioners.

**Fig.2:**
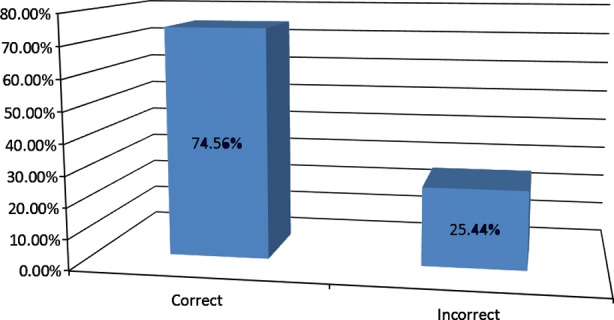
Basic concepts and management skills of GPs regarding Ramadan and diabetes.

**Fig-3:**
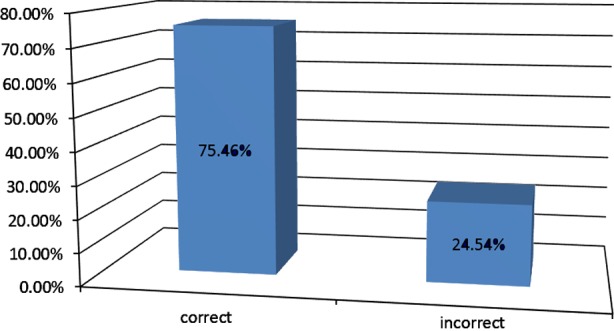
Physical activity and diet related Ramadan specific knowledge of GPs.

**Fig-4:**
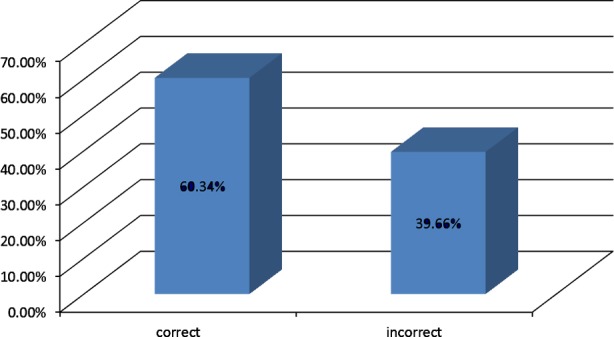
Knowledge of GPs related to treatment modification in patients with diabetes during.

About 16.73% (+ 2.05%) of the GPs responded that no adjustment in “drug dosage” is needed in people with diabetes during Ramadan. While more than 80% responded correctly regarding alteration in “timing” of medications (Table-I).

**Table-I:** Responses of GPs regarding Ramadan specific knowledge.

***A. Basic concepts and management skills of GPs regarding Ramadan and diabetes.***			

*Questions*	*Correct*	*Incorrect*	*Don’t know*

1. Patients with diabetes should never fast	81.4%	16.94%	1.66%
2. Taking insulin injection during fasting breaks the fast?	68.04%	25.72%	6.24%
3. Sehri can be skipped during Ramadan?	92.8%	6.4%	0.8%
4. Checking blood sugar levels during fasting is prohibited?	78.4%	19.02%	2.44%
5. If BSL is around 70mg/dl in early hours of fasting, it is advised to immediately consult the physician or break the fast?	64.59%	32.08%	3.33%
6. Optimum blood sugar level during fasting is, • 60-120mg/dl, • 100-200mg/dl • 200-300mg/dl?	51.83%	48.17%	-
7. If one and half hour is left for iftar and BSL is around 75mg/dl, appropriate step would be: • stop all activities • immediately break the fast • Continue with routine work?	74.89%	25.11%	-
8. If BSL is above 300mg/dl, patient is advised to, • consult the physician, • break the fast, • Do not know?	84.48%	15.52%	-

***B. Physical activity and diet related Ramadan specific knowledge of GPs***			

9. Strenuous exercise/physical activity should be avoided during fasting?	83.67%	13.88%	2.45%
10. Is tarawih prayer an alternative of exercise during Ramadan?	77.55%	21.22%	1.23%
11. Complex CHO should be taken at iftar and simple CHO should be taken at sehri?	64.19%	26.33%	9.48%
12. Lemonade with artificial sweetener and lassi can be taken in iftar?	76.44%	19.42%	4.14%

***C. Knowledge of GPs related to treatment modification in patients with diabetes during Ramadan***			

13. Best option for timings of medication during Ramadan is, • continue same as before Ramadan, • morning dose at iftar and night dose at sehri, • No treatment needed, only hold fast is the best option?	85.72%	14.28%	-
14. Patients on metformin BD dosage should, • continue their dose on BD basis, • take only OD dose at iftar, • Stop metformin?	62.98%	37.02%	-
15. Patients taking TDS dose of metformin should, • take total dose at iftaar on OD basis • take 1/3rd at sehri and 2/3rd at iftaar • take ½ at sehri and half at iftaar.	63.78%	36.22%	-
16. Patient on sulfonylurea as OD dose should • reduce dose to 75% of actual • continue same dose • Stop sulfonylurea during Ramadan?	58.68%	41.32%	-
17. Patient on sulfonylurea BD dose should, • reduce morning dose to 75% and night dose to 50% • reduce morning dose to 50% and night dose to 75%, • no change required	40.08%	59.92%	-
18. No adjustment in drug dosage and timings is recommended for fasting patients with diabetes?	81.22%	16.73%	2.05%
19. Patient on single basal insulin and oral combined treatment regimen should have basal insulin dose reduced by 30% of actual dose?	63.63%	29.33%	7.04%
20. Patient on sulfonylurea combined with basal bolus insulin regimen need no change in their usual dose of sulfonylurea?	61.38%	26.82%	11.8%
21. Patients on pre-mixed insulin should continue their pre-Ramadan dose?	60.16%	33.33%	6.51%
22. Patients on 70/30 insulin regimen should reduce their morning dose to 75% and night dose to 50% of the actual dose?	68.54%	26.20%	5.26%
23. Reduced morning dose of 70/30 should be used at sehri?	28.74%	67.20%	4.06%
24. Patients on R and N regime need no reduction in evening pre-Ramadan dose?	47.08%	40.41%	12.51%
25. Patients on R and N regimen need 50% reduction in pre-Ramadan evening dose taken at sehri?	62.34%	21.75%	15.91%

## DISCUSSION

This study, on assessment of baseline knowledge of general practitioners across the country, found several inadequacies among doctors regarding management of diabetes in fasting patients during the month of Ramadan. Almost one third of the study population gave incorrect answers, thus highlighting the need of health professional’s education in this specific field of diabetes.

In our study, we have noted that a sizeable proportion of general practitioners have misconceptions regarding fasting in patients with diabetes. 1/4^th^ of the GPs thought that people with diabetes should never fast which is contrary to the evidence in the literature.^6^ One third of the GPs believed that taking insulin injection during fasting will break the fast. This myth is common among patients with diabetes but this survey has highlighted misconceptions among our GPs too. This also points towards poor religious knowledge. Although majority of the doctors were of the opinion that Sehri should not be skipped but small number of doctors gave incorrect answer which can lead to complications especially in their patients with type 1 diabetes. Monitoring of blood sugar levels during fasting is important and it has been shown that active glucose monitoring plays an important role in the documentation of hypo and hyperglycemia episodes during fasting.^7^,^8^ Checking blood sugar levels on development of symptoms (hypo/hyper) can enable the patients to break the fast if the limits are crossed but one third of the GPs did not know about the proper action that should be taken in case of development of hypoglycemia. Around 1/4^th^ of the GPs did not know what to advise if patients develop hyperglycemia during fasting.

Maintaining a balanced diet during Ramadan is also one of the cornerstones of safe fasting in people with diabetes.^2^,^9^ During Ramadan the intake of carbohydrates should be well distributed and utilization of large amounts of food rich in carbohydrates and fats should be avoided specially at iftar.^2^,^10^ The baseline knowledge of GPs regarding distribution of simple and complex carbohydrates during Ramadan was not up to the mark in around more than one third of the GPs.

Excessive physical activity may lead to higher risk of hypoglycemia during fasting.^9^,^10^ All people with diabetes who wish to fast should be counseled properly to maintain normal levels of physical activity during fast to avoid any complication. The extra prayers (Tarawih) that are offered during Ramadan after Iftar consist of excessive physical activity. So it should be considered as a part of routine exercise programme.^2^ Almost 1/5^th^ of the GPs did not have proper knowledge regarding physical activity during Ramadan. This issue needs consideration to avoid hazardous outcomes during fasting.

Almost 40% of the GPs had improper knowledge regarding dose adjustments and timings of medication during Ramadan. More inaccuracies were found in the adjustment of dose of regular and NPH insulin and sulphonylureas. Medication taken without adjustment of dosage and timing alteration can lead to serious complications like hypo or hyperglycemia as we have shown in our Ramadan prospective diabetes study.^7^

Few studies have been conducted to evaluate the knowledge, altitude and practices of general practitioners regarding management of diabetes during Ramadan^11^,^12^,^13^ but to the best of our knowledge, no such studies have been conducted so far in Pakistan. This study mainly focused on the base line knowledge and the medical practices of GPs practicing in different areas of Pakistan and was a part of Ramadan education program that was designed to educate doctors who were giving medical care to patients with diabetes.

The present study has not looked into the impact of education and training of GPs ie, posttest assessment was not carried out in this group of GPs. Further studies are needed to ascertain the utility of training workshops in improving the knowledge and actual change in practices of general practitioners dealing with fasting patient with diabetes across the country.

## CONCLUSION

General practitioners are the backbone of our health care system. Training workshops and CMEs for GPs seems compulsory for better outcomes in fasting patients with diabetes. Medical curriculum should include chapter on Ramadan and diabetes to equip doctors with required knowledge and training to meet the challenge of safe fasting in their patients with diabetes.
